# Heat Stress Assessment Using Multiple Thermal-Comfort Indices and Its Impact on the Reproductive Performance of Sows and Their Offspring in a Temperate Climate

**DOI:** 10.3390/vetsci13030270

**Published:** 2026-03-15

**Authors:** Maria Chavez-Flores, Abel Villa-Mancera, José Manuel Robles-Robles, Jaime Olivares-Pérez, Agustín Olmedo-Juárez, Alejandro Córdova-Izquierdo, Roberto González-Garduño, José Luis Ponce-Covarrubias, Nallely Rivero-Perez, Felipe Patricio, Adrián Muñoz-Cuautle, Samuel Ortega-Vargas

**Affiliations:** 1 Facultad de Medicina Veterinaria y Zootecnia, Benemérita Universidad Autónoma de Puebla, Tecamachalco 75482, Puebla, Mexico; cf224462162@alm.buap.mx (M.C.-F.); manuel.roblesr@correo.buap.mx (J.M.R.-R.); felipe.patriciomtz@correo.buap.mx (F.P.); adrian.munozcuautle@correo.buap.mx (A.M.-C.); samuel.ortega@correo.buap.mx (S.O.-V.); 2 Programa de Maestría en Producción Animal Sostenible, Benemérita Universidad Autónoma de Puebla, Tecamachalco 75460, Puebla, Mexico; 3 Unidad Académica de Medicina Veterinaria y Zootecnia, Universidad Autónoma de Guerrero, Ciudad Altamirano 40662, Guerrero, Mexico; jaimeolivares@uagro.mx; 4 Centro Nacional de Investigación Disciplinaria en Salud Animal e Inocuidad (CENID SAI-INIFAP), Jiutepec 62550, Morelos, Mexico; olmedo.agustin@inifap.gob.mx; 5 Departamento de Producción Agrícola y Animal, Universidad Autónoma Metropolitana, Unidad Xochimilco, Ciudad de México 04960, Mexico; acordova@correo.xoc.uam.mx; 6 Unidad Regional Universitaria Sursureste, Universidad Autónoma Chapingo, Teapa 86800, Tabasco, Mexico; rgonzalezg@chapingo.mx; 7 Escuela Superior de Medicina Veterinaria y Zootecnia No. 3, Universidad Autónoma de Guerrero, Técpan de Galeana 39086, Guerrero, Mexico; jlponce@uagro.mx; 8 Área Académica de Medicina Veterinaria y Zootecnia, Instituto de Ciencias Agropecuarias, Universidad Autónoma del Estado de Hidalgo, Pachuca 42039, Hidalgo, Mexico; nallely_rivero@uaech.edu.mx

**Keywords:** heat stress, sow reproduction, thermal comfort indices, temperature–humidity index (THI), stillbirth piglets, mummified fetuses, climate change adaptation

## Abstract

This study aimed to evaluate and compare the effects of HS on sow reproductive performance using 11 different thermal indices calculated from environmental data in a temperate climate. Reproductive records, including live-born piglets, stillbirths, and mummified fetuses per litter, were obtained using farm management software. The overall means for live-born piglets, stillbirths, and mummified fetuses per litter from January to December 2023 were 13.7, 1.06, and 0.45, respectively, whereas the temperature, relative humidity, and air velocity were 24.08 °C, 63.70%, and 3.2 m/s, respectively. Temperature–humidity index (THI): THI2 and THI6 were significantly associated with the number of live-born piglets (*p* < 0.001) and mummified fetuses (*p* < 0.05) per litter, with optimal reproductive outcomes observed at values below 74. THI1 was significantly associated with the number of stillborn piglets (*p* < 0.05). Based on the Akaike information criterion (AIC) values, THI6 provided the best overall fit for evaluating the effects of HS on reproductive performance. Reproductive outcomes in sows respond to trait-specific thermal signals. THI2, THI6, and THI1 are recommended for assessing HS-related reproductive outcomes in sows raised under temperate climatic conditions.

## Simple Summary

This study examined the effects of heat stress (HS) on sow reproduction using 11 different thermal indices in a temperate climate area. The number of live-born piglets, stillbirths, and mummified fetuses per litter were recorded in the farm management software. From January to December 2023, we recorded 13.7 live-born piglets per litter, 1.06 stillborn piglets per litter, and 0.45 mummified fetuses per sow. The mean temperature was 24.08 °C, humidity 63.70%, and air speed 3.2 m/s. The temperature–humidity index (THI) showed that THI2 and THI6 were linked to more live-born piglets and fewer mummified fetuses, with optimal results below a THI of 74. THI1 was associated with an increased number of stillborn piglets. THI6 was the best thermal comfort index for studying the effects of HS on reproductive performance in this study. Sow reproductive outcomes are influenced by specific thermal signals. THI2, THI6, and THI1 are suggested for assessing HS-related reproductive outcomes in sows in temperate climates. Integrating these indices with physiological biomarkers, such as cortisol levels or infrared thermography, could improve reproductive efficiency and animal welfare.

## 1. Introduction

HS occurs when the balance between heat production and dissipation in pigs is disrupted, and it can be affected by several environmental factors [1]. In porcine species, the selection of more productive genetic lines has increased the metabolic heat in animals [2], which has reduced fertility and caused embryonic deaths in early pregnancy, thereby reducing the subsequent farrowing rate and litter size [3].

In sows, temperatures above 24–25 °C induce HS, which can be measured using the temperature–humidity index (THI), leading to oxidative stress in sows, resulting in a decrease in farrowing events and piglets born and weaned, as well as an increase in piglet mortality [4,5]. In temperate climates, THI is a reliable indicator of HS, affecting pregnancy and farrowing rates, as higher THI values are associated with decreased reproductive performance [6].

In contrast, the thermoneutral zone for piglets is typically between 34 and 36 °C, where they maintain their body temperature without additional energy expenditure and experience caloric stress, particularly below 20 °C or above 38 °C [7,8]. High ambient temperatures and relative humidity lead to higher piglet mortality and mummification rates, especially in highly prolific sows, due to inadequate colostrum intake and prolonged farrowing durations [9]. Gestational exposure to HS can modulate both the number of live-born piglets and increase stillbirth incidence, with lasting effects on offspring health and productivity [10].

HS causes severe economic losses in sows due to reductions in reproduction, growth, and overall productivity. Economic losses due to HS in the United States swine industry have been estimated at US$ 299 million per year [11]. The predicted global mean rise in temperature by 0.3 and 0.7 °C for the period between 1986 and 2035 [12] reflects productivity challenges for the pig industry due to HS complications, causing the pork industry to face productivity challenges due to HS [11]. HS in pigs, exacerbated by climate change, presents significant challenges to pig productivity and welfare in the swine industry. By the end of the century, the likelihood of extreme HS is anticipated to increase in pigs, especially in tropical and some temperate zones, which would undermine the sustainability of outdoor livestock systems [13].

The HS assessment allows for the detection of environmental components that guide temperature control in the production units. Several equations have been developed to calculate the THI, which are adjusted for different indices designed to account for individual physiological reactions. THI has been extensively utilized across various species, including cattle, buffaloes, pigs, and poultry. The most commonly utilized thermal indices include the enthalpy (H), equivalent temperature index of sows (ETIS), effective temperature (ET), black globe–humidity index (BGHI), and THI [14]. This study aimed to investigate the effect of HS on the reproductive performance of sows by utilizing various thermal comfort indices derived from environmental factors in a temperate climate and to analyze the goodness-of-fit among the ETIS, ET, BGHI, and THI, equations.

## 2. Materials and Methods

### 2.1. Study Area and Sows

The study was conducted in the central Mexican state of Puebla from January to December 2023 on a farm with 1200 Camborough sows. The production site is located in the Tecamachalco district, characterized by a temperate climate (Cw) based on the modified Köppen climate classification system proposed by Garcia [15]. Rainfall averages 586.3 mm per year, and the mean annual temperature is 22.4 °C (minimum: 11.3 °C), and the rainy season extends from May to September [16]. Pig Vision farm management software (Agrovision BV, Denver, The Netherlands) was used to obtain the reproductive records. These data included the identity of the sows and variables, such as the number of live piglets born, stillborn piglets, and mummified fetuses.

### 2.2. Semen Samples and Artificial Insemination (AI)

Semen samples were obtained from a local commercial artificial insemination center featuring boars of the PIC-337 and PIC-410 breeds aged 2–3 years. Two weekly ejaculations were facilitated using the gloved-hand technique, consistently executed by a single operator. Semen quality was evaluated using a sperm analyzer(Ultimate Sperm Analyzer, version 12.21; Hamilton Thorne Biosciences, MA, USA) to accurately measure motility and viability. Fresh semen was diluted following the Beltsville technique (the final volume of which was 80 mL containing a total of 3 × 10^9^ viable spermatozoa) and refrigerated at 17 °C for a period not exceeding 48 h. Subsequently, heat detection was conducted by exposing the boars. Animals identified as being in estrus were subjected to artificial insemination at 12, 24, and 36 h using fresh semen dosages administered via an intrauterine catheter (Goldenpig; IMV Technologies, L’Aigle, France). Pregnancy was confirmed using ultrasonography (Preg-Tone, Renco Corporation, Minneapolis, MN, USA) 35 and 70 days after artificial insemination. Abortion instances were documented throughout the gestational period. Sows were moved into the farrowing pens 3 to 5 days prior to the expected farrowing.

### 2.3. Meteorological Data and Temperature Indices

Climatic variables such as temperature (°C), air velocity (km/h), and relative humidity (%) were registered from January to December 2023 using a meteorological network operated by EMASCONAGUA, which is approximately 3 km away from the farm location (https://smn.conagua.gob.mx/es/). We published equations for a number of thermal comfort indices (Table 1) in the context of heat-stress response. The dew point temperature (T_dp_), wet-bulb temperature (T_wb_), and black globe temperature (T_g_) were calculated according to the equations suggested by Cao et al. [17].

### 2.4. Statistical Analyses

Continuous reproductive variables, including the number of piglets born alive, stillborn, and mummified fetuses per litter, were analyzed using linear mixed models to account for the longitudinal nature of the data and repeated measurements. The fixed effects in the models consisted of the mean THI calculated for each gestational period (piglets born alive: days 0–35, mummified piglets 0–50, and stillborn pigs 102–110). To account for the non-independence of observations and individual biological variance, identity of sow was included as a random effect. Means were compared using Tukey’s test, with significance defined as *p* < 0.05. The IBM SPSS 25 software package (SPSS Inc., Chicago, IL, USA) for Windows was used for analytical statistics. Akaike information criterion (AIC) was used to determine the goodness-of-fit for thermal comfort index.

## 3. Results

### 3.1. Climatic Factors and Reproductive Performance

**Figure 1:**
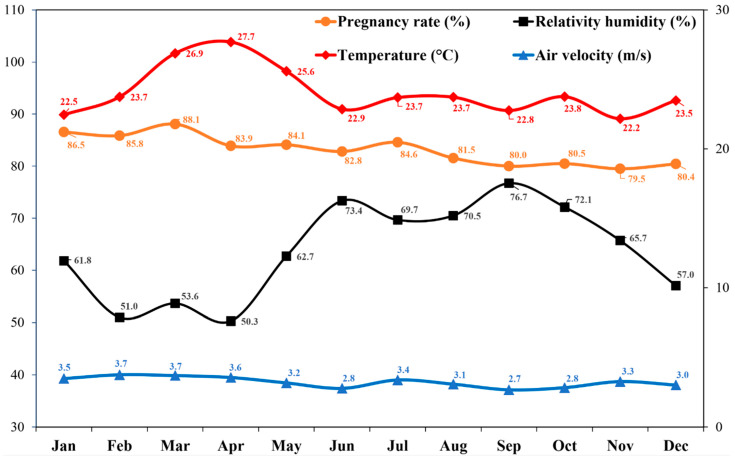
Climate parameters and pregnancy rates during the study period in a temperate climate were summarized as monthly values.

The meteorological station trends over the study period, as well as the average monthly temperature, humidity, wind speed, and gestation rates, are shown in Figure 1. The mean ambient temperature was 24.08 °C, relative humidity was 63.70%, and wind speed was 3.2 m/s. The overall gestation rate was 83.1%. The highest temperatures were recorded in April, and the lowest temperatures were recorded in November. With respect to relative humidity, the highest values were observed in September and the lowest in April. The highest and lowest gestation rates were recorded in March and November, respectively.

### 3.2. Thermal Indices and Reproductive Efficacy

**Figure 2:**
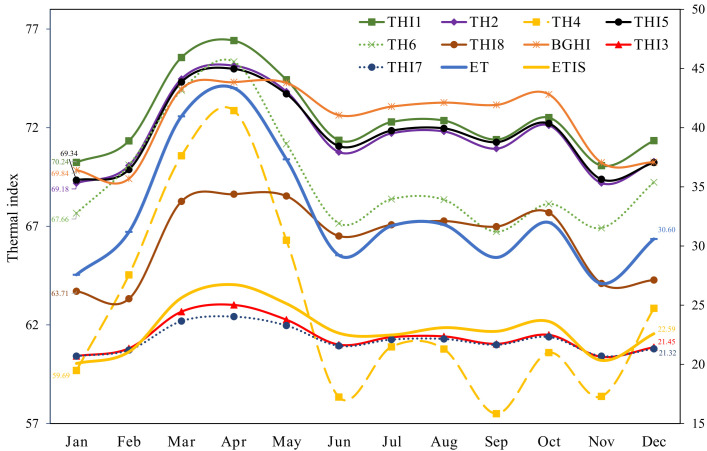
Mean monthly data for multiple thermal-comfort indices observed over the course of the study in a commercial pig farm located in central Mexico. ETIS: equivalent temperature index; ET: effective temperature; BGHI: black globe–humidity index; THI: temperature–humidity index of sows.

Figure 2 illustrates the trends in the mean thermal indices during the gestation periods throughout the study. The minimum values for these indices were observed in January, except for THI4 and THI6 in September, whereas ET was the lowest in November. In contrast, the highest values were observed in April. Subsequently, THI1, THI2, THI5, THI7, THI8, and BGHI progressively diminished until October, whereas THI3, THI4, THI6, and THI7 decreased from June to July.

### 3.3. Impact of Thermal Comfort Indices on Reproductive Performance

Table 2 summarizes the associations between reproductive parameters and various thermal comfort indices, categorized by specific threshold divisions. The highest percentage of live piglets was observed for THI2 and THI6 levels of less than 74, which corresponded to suitable and mild-stress indices. Statistically significant differences were found among THI2 (*p* < 0.05), THI6 (*p* < 0.001), and the various threshold divisions and live-born piglets. However, THI1, THI3, THI4, THI5, THI7, THI8, BGHI, ET, and ETIS did not significantly affect the live piglet rate (*p* > 0.05).

The average number of mummified piglets per litter was higher for THI2 and THI6, with values below 74, corresponding to the categories of suitable stress conditions. In contrast, mummified fetuses had a lower incidence in THI2 and THI6 with values of ≥74–<78. Our experiments demonstrated substantial differences in the farrowing rate among THI2 (*p* < 0.05) and THI6 (*p* < 0.001) and the farrowing rate. No differences were observed between THI1, THI3, THI4, THI5, THI7, THI8, BGHI, ET, ETIS, and mummified piglets (*p* > 0.05).

For stillborn piglets exposed to thermal comfort stress, the abortion rate was constantly higher for THI1 values. Conversely, the lowest abortion rates occurred at THI1 values between ≥69 and ≤73, representing thermal stress. There were statistically significant differences between the THI and number of dead piglets at birth (*p* < 0.05); however, no significant correlations were noted between THI2, THI3, THI4, THI5, THI6, THI7, THI8, BGHI, ET as well as the value of ETIS. The Tukey test was performed to compare the significant differences in different thermal comfort indices as well as reproductive parameters. Based on 95% confidence level, the Tukey test indicated substantial disparities in the overall mean values for THI2 and THI6 concerning live and mummified piglets per litter, as well as THI1 values on stillborn piglets between the different thermal comfort thresholds.

THI1 values in stillborn pigs under thermal comfort stress were consistently associated with higher abortion rates. Similarly, THI1 values of >69 and <73 (thermal stress) showed the lowest rates of mortality rates. The THI and the number of dead piglets at birth were shown to differ statistically significantly (*p* < 0.05), but not the correlations between the THI21, THI3, THI4, THI5, THI6, THI7, THI8, BGHI, ET, and ETIS values. Differences among thermal comfort indices and reproductive parameters were analyzed using Tukey’s test. At the 95% CI level, significant differences in the overall mean were observed for THI2 and THI6 values with respect to live piglets per litter and mummified piglets per litter and those of THI1 for the stillborn piglets among different thermal comfort classes.

Five of the eleven comfort indices examined in this study were selected for their statistical significance to evaluate the goodness of fit of the data using different THI or ET equations, as indicated by the lowest values of the AIC. The corresponding AIC values of the THI6 for live born pigs, mummified fetuses and stillborn pigs were 8980.8, 5818.3 and 8296.9, respectively. These data suggest that THI6 is the index of HS which best reflects in these animals.

## 4. Discussion

The impact of HS on reproductive parameters such as the number of live-born offspring, mummies, and stillborn piglets, in inseminated sows in temperate climatic conditions were evaluated. The observed litter size of live piglets is in agreement with previous reports from temperate regions and under usual breeding conditions, in which an average of 12–14 live-born piglets are routinely reported [28,29].

Our findings are in partial agreement with those of previous studies that reported the detrimental effects of HS on the reproductive performance of sows of different breeds. Pregnant sows are particularly prone to this syndrome because of their relatively large body mass and intense metabolic demands, often leading to reduced pregnancy success and lower offspring viability [10]. Brito et al. [30] further demonstrated that tropical pigs exposed to heat have significantly lower reproductive performance, with increased stillbirths and reduced litter size. Similarly, we found very high counts of live piglets in the temperate region, and it is possible that the intensity and duration of HS events also affected the results.

The concurrent observation of 1.06 stillborn piglets per litter suggests that HS affects neonatal survival rather than overall conception rate [31]. Stillbirths are often the result of acute heat episodes occurring during late pregnancy (102–110 days) [28,32]. The drop in productivity affirms the idea that HS is most detrimental at late gestation and farrowing, when growing fetuses require more oxygen from their mother, which, in turn, increases the body heat output.

The rate of stillbirths found in this study is equivalent to that which has previously been reported when sows are exposed to mild HS [28,33], and it is lower than those recorded during severe exposure or chronic exposure to high temperatures (>30 °C), when stillbirths can exceed two piglets per litter [11]. This gap probably reflects differences in the magnitude and duration of heat exposure, as well as the practices of adaptive management typical of temperate areas. Our results, however, would indicate that there is no evident defined thermal safety for the gestating mother, since subacute HS can compromise placental efficiency and fetal oxygenation [34].

On average, there were 0.45 ± 0.83 mummified piglets per litter, indicating that the thermal challenge in temperate zones may be more episodic than constant. Mummification requires a sustained insult during the first five weeks of pregnancy to cause fetal death without abortion [35]. Increased fetal mummification has been associated with early embryonic mortality resulting from deficient uterine blood flow, oxidative stress, and endocrine disruption, as reported in prior studies [36,37]. Some reports suggest increased mummy rates under extremely hot conditions; others find only small increases similar to those described here, especially among well-managed herds [38]. It is remarkable that our mummified piglet rate was relatively low compared to the range of 0.95 ± 1.1 described by [39] for heat-stressed sows during pregnancy. The variation in this effect suggests that mummification may be more sensitive to the duration of heat exposure than the average ambient temperature, which agrees with the developmental stage-specific sensitivity models.

The high standard deviations of 1.40 and 0.83 for stillborns and mummies, respectively, revealed a high management inadequacy in the farm system. This means that while some sows are handled very well, others fail. This requires the use of precision livestock farming techniques. Using sensors to monitor vaginal temperature and respiratory rate sensors facilitates the identification of animals at high risk during periods of heatwaves, thus providing focused efforts, such as oxytocin supplementation or manual assistance in prolonged farrowing. Likewise, this variability may be due to recorded ambient temperatures that may mask microclimate variations within the farrowing house, whereas the actual heat perceived by the sow at the floor level (where ventilation may be stagnant) could be significantly higher.

The evolution of THI-based thermal comfort indices throughout the year further illustrates the sensitivity of swine reproductive outcomes to compounded climate stressors. The majority of indices peaked in April and reached their lowest values in January, following the pattern of ambient temperature; however, certain indices (e.g., THI3, THI4, THI6, and THI7) declined as early as June–July. These differences reflect the varied weighting of humidity, vapor pressure, and radiant heat within each equation, corroborating the findings of da Fonseca et al. [40], who reported that not all THI formulations respond to seasonal climate transitions uniformly. This divergence also supports the theoretical work of Collier and Gebremedhin [41], who emphasized the complexity of evaluating HS using indices originally designed for cattle and humans.

The 30 °C developed by Thom in 1958 to estimate stress levels in humans, has been frequently used to assess HS in pigs and cattle due to its simplicity and practicality [14,42]. In contrast, THI6 was developed by the National Oceanic Atmospheric Administration [23] as a standardized tool for predicting and mitigating HS in livestock.

In the present study, the strong association of THI2 and THI6 values around 74 with the highest number of live-born piglets reinforces prior evidence that THI thresholds between 72 and 74 mark the onset of thermal stress in gestating sows [43,44]. The highly significant differences (*p* < 0.001) observed for these indices suggest that incorporating humidity and temperature interaction terms enhances their predictive capacity. In contrast, the absence of strong associations for THI3, THI4, THI5, THI7, THI8, BGHI, ET, and ETIS is consistent with the findings of Cao et al. [17], who reported considerable inconsistency among popular thermal indices when applied to pigs in field settings.

The observed pattern in mummified piglets, where the highest incidence rates were recorded at THI2 and THI6 values ranging from 74 to 78, indicates that prolonged exposure to moderate stress may be more damaging than short-term extremes. This aligns with the concept of cumulative thermal load described by St-Pierre et al. [11].

Conversely, THI1, developed by Thom [18], was the most predictive index for stillborn piglets in the present study. This index has been used to compare the thermal demand in different regions and at different times of the year for both buildings and livestock production. Likewise, this thermal comfort index provides information for planning the environment of the facilities. In our study, stillbirths peaked at THI1 values of 65–69, corresponding to an intermediate thermal stress. This supports the distinction proposed by Borges et al. [45], who argued that stillbirth is more sensitive to acute intrapartum heat load than to prolonged stress during gestation. Together, these findings suggest that different reproductive traits are mediated by distinct physiological mechanisms and, therefore, respond to different thermal signals.

The statistical significance of THI1, THI2, and THI6 underscores the importance of selecting indices tailored to specific reproductive outcomes. Similar findings in cattle suggest that different THI equations predict different physiological responses [46], reinforcing the need for species-specific calibrations of the equations. Despite the robustness of the statistical models, the study relied on climatic data from a weather station, which may not perfectly reflect the microclimate inside the gestation facilities. Accurate monitoring of the microclimate is essential for optimizing animal welfare and productivity [47].

Contradictions arise when comparing gestation rates across various climates. Knox [48] reported minimal seasonal variation in temperate regions, whereas our study found significant declines during the colder months. This discrepancy may be explained by differences in housing systems; climate-controlled facilities mitigate cold stress, whereas semi-open housing exposes sows to extreme temperatures.

## 5. Conclusions

This study is the first to comparatively evaluate different thermal comfort indices and their values with respect to the environmental factors affecting the reproductive performance of sows in temperate climates. These findings emphasize the need to assess the effectiveness of current HS abatement strategies and whether they require further improvements. Significant negative effects on reproductive performance were observed when the temperature and THI values reached certain levels. Among the thermal indices evaluated, THI2, THI6, and THI1 demonstrated the best fit for analyzing HS in sows reared in temperate climates. Future studies are required to improve our knowledge of the different thermal comfort indices adapted to each climatic region and housing system. This should also involve daily climatic data and the integration of genetic and nutritional factors.

## Figures and Tables

**Table 1:** Equations pertaining to various thermal comfort indices are accompanied by specific threshold delineations.

Thermal-Comfort Indices	THI Limit	Species Utilized	Source
THI1 = T + 0.36 × T_wb_ + 41.5	Thermal comfort <61–≤65 Intermediate > 65–≤ 69 Thermal stress > 69–≤ 73	General	Thom [18]
THI2 = 0.8 × T + ((RH/100) × (T − 14.3)) + 46.4	Suitable < 74 Mild ≥ 74–< 78 Moderate ≥ 78–< 82 Severe ≥ 82	General	Thom [19]
THI3 = 0.65 × T + 0.35 × T_wb_	Heat stress ≥ 28 None stress	Swine	Ingram [20]
THI4 = T° − (0.55 − 0.0055 × RH) × (T° − 58)	-	General	Kelly and Bond [21]
THI5 = 0.72 × T + 0.72 × T_wb_ + 40.6	Moderate ≤ 75–< 78 Severe ≥ 83	Dairy cattle	Maust et al. [22]
THI6 = (1.8 × T + 32) − (0.55 × (RH/100)) × ((1.8 × T + 32) − 58)	Suitable ≤ 74 Mild > 74–≤ 78 Moderate > 78–≤ 84 Severe > 84	Dairy cattle, swine	NOAA [23]
THI7 = T − (0.55 − 0.0055 × RH) × (T − 14.5)	-	Dairy cattle, swine	NWSCR [24]
THI8 = 0.27 × T + 1.35 × T_wb_ + 34.07	-	Swine, poultry	Fehr et al. [25]
BGHI = T_g_ + 0.36 × T_dp_ + 41.5	-	Dairy cattle	Buffington et al. [26]
ET = T + 0.0015 × (RH − 50) × T + (−1.0 × 42 − T × (v^0.66^ − 0.2^0.66^))	-	Swine	Bjerg et al. [27]
ETIS = T + 0.0006 × (RH − 50) × T − 0.3132 × u^0.6827^ × (38 − T) − 4.79 × (1.0086 × 38 − T) + 4.8957 × 10^−8^ × ((38 + 273.15)^4^ − (T + 273.15)^4^)	Suitable < 33.1 Mild ≥ 33.1–< 34.5 Moderate ≥ 34.5–< 35.9 Severe ≥ 35.9	Sow	Cao et al. [14]

BGHI: black globe–humidity index; ET: effective temperature; ETIS: equivalent temperature index of sows. T: dry bulb temperature (°C); T_wb_: wet bulb temperature (°C); RH: relative humidity index (%); T°: dry bulb temperature (°F); T_g_: black globe temperature (°C); T_dp_: dew point temperature (°C); u, v: air velocity (m/s). Table adapted from Cao et al. [17].

**Table 2:** Different thermal comfort indices and limit thresholds to evaluate the effect of HS on sows in a temperate climate in Mexico.

Thermal-Comfort Indices	*n*	Live Born	*p*-Value	AIC	Mummies	*p*-Value	AIC	Stillborn	*p*-Value	AIC
THI1										
Thermal comfort <61–≤65	-	-			-			-		
Intermediate > 65–≤ 69	-	-			-			0.77 ± 3.25 ^a^	0.036	8296.9
Thermal stress > 69–≤ 73	2359	13.7 ± 1.6		9010.8	0.45 ± 0.83	-	5827.0	1.08 ± 1.26 ^b^		
THI2										
Suitable < 74	1923	13.7 ± 1.4 ^a^	<0.001	8988.2	0.46 ± 0.85 ^a^	0.047	5827.4	1.07 ± 1.43	0.538	8302.0
Mild ≥ 74–< 78	436	14.1 ± 2.09 ^b^			0.37 ± 0.72 ^b^			1.02 ± 1.19		
Moderate ≥ 78–< 82	-	-		-	-			-		
Severe ≥ 82	-	-		-	-			-		
THI3										
Heat stress ≥ 28	2352	13.7 ± 1.6	0.417	9009.3	0.45 ± 0.83	-	5827.0	1.06 ± 1.40	-	8299.3
None stress	7	14.2 ± 1.1								
THI4										
	2359	13.7 ± 0.03		9010.8	0.45 ± 0.83	-	5827.0	1.06 ± 1.40	-	8299.3
THI5										
Moderate ≤ 75–< 78	2359	13.7 ± 0.03		9010.8	0.45 ± 0.83	-	5827.0	1.06 ± 1.40	-	8299.3
Severe ≥ 83	-	-			-					
THI6										
Suitable ≤ 74	2151	13.7 ± 1.4 ^a^	<0.001	8980.8	0.47 ± 0.85 ^a^	<0.001	5818.3	1.07 ± 1.42	0.419	8301.4
Mild > 74–≤ 78	208	14.3 ± 2.5 ^b^			0.26 ± 0.57 ^b^			0.99 ± 1.12		
Moderate > 78–≤ 84	-	-			-					
Severe > 84	-	-			-			-		
THI7	2359	13.7 ± 1.6	-	9010.8	0.45 ± 0.83	-	5827.0	1.06 ± 1.40	-	8299.3
THI8	2359	13.7 ± 1.6	-	9010.8	0.45 ± 0.83	-	5827.0	1.06 ± 1.40	-	8299.3
BGHI	2359	13.7 ± 1.6	-	9010.8	0.45 ± 0.83	-	5827.0	1.06 ± 1.40	-	8299.3
ET	2359	13.7 ± 1.6	-	9010.8	0.45 ± 0.83	-	5827.0	1.06 ± 1.40	-	8299.3
ETIS										
Suitable < 33.1	2352	13.7 ± 1.6	0.417	9009.3	0.45 ± 0.83	-	5827.0	1.06 ± 1.40	-	8299.3
Mild ≥ 33.1–< 34.5	7	14.2 ± 1.1			-			-		
Moderate ≥ 34.5–< 35.9	-	-			-			-		
Severe ≥ 35.9	-	-			-			-		

Different letters within a column (for each index) indicate statistically signiﬁcant differences between the different levels of an index (*p* < 0.05). AIC, Akaike’s information criterion; ETIS: equivalent temperature index; ET: effective temperature; BGHI: black globe–humidity index; THI: temperature–humidity index of sows.

## Data Availability

The original contributions presented in this study are included in the article. Further inquiries can be directed to the corresponding author.

## Abbreviations

The following abbreviations are used in this manuscript:

THI	Temperature–humidity index
HS	Heat stress
BGHI	Black globe–humidity index
ET	Effective temperature
ETIS	Equivalent temperature index of sows
H	Enthalpy
AI	Artificial insemination
AIC	Akaike information criterion
T_dp_	Dew point temperature
T_wb_	Wet-bulb temperature
T_g_	Black globe temperature
